# Lived Experience of Iranian Family Caregivers of Tubercular Patients: A Qualitative Study*

**DOI:** 10.17533/udea.iee.v40n3e02

**Published:** 2023-02-08

**Authors:** Zinat Mohebbi, Raziye Dehbozorgi, Giti Setoodeh, Marzieh Momennasab, Naval Heydari, Maryam Shaygan

**Affiliations:** 1 Assistant Professor, Department of Nursing. School of Nursing and Midwifery, Shiraz University of Medical Sciences, Shiraz, Iran. Email: mohebbi04@yahoo.com Shiraz University Department of Nursing School of Nursing and Midwifery Shiraz University of Medical Sciences Shiraz Iran mohebbi04@yahoo.com; 2 Ph.D. Candidate. Nursing and Midwifery School, Isfahan University of Medical Sciences, Isfahan, Iran. Email: rdehbozorgi110@gmail.com University of Isfahan Nursing and Midwifery School Isfahan University of Medical Sciences Isfahan Iran Email: rdehbozorgi110@gmail.com; 3 Assistant Professor, Department of Mental Health and Psychiatric Nursing. Shiraz University of Medical Sciences, Shiraz, IranEmail: setoodeh@sums.ac.ir Shiraz University Department of Mental Health and Psychiatric Nursing Shiraz University of Medical Sciences Shiraz Iran setoodeh@sums.ac.ir; 4 Associate Professor, Department of Nursing. Shiraz University of Medical Sciences, Shiraz, IranEmail: momennasab@sums.ac.ir Shiraz University Department of Nursing Shiraz University of Medical Sciences Shiraz Iran momennasab@sums.ac.ir; 5 Ph.D. Candidate, Student Research Committee, Community Based Psychiatric Care Research Center, Department of Nursing. Shiraz University of Medical Sciences, Shiraz, Iran Email: navalheydari@gmail.com. Corresponding author. Shiraz University Department of Nursing Shiraz University of Medical Sciences Shiraz Iran navalheydari@gmail.com.; 6 Associate Professor, Community Based Psychiatric Care Research Center, Department of Nursing. Shiraz University of Medical Sciences, Shiraz, Iran Email: m2620.shaygan@gmail.com Shiraz University , Department of Nursing Shiraz University of Medical Sciences Shiraz Iran m2620.shaygan@gmail.com

**Keywords:** caregivers, hermeneutics, qualitative research, tuberculosis., cuidadores, hermenéutica, investigación cualitativa, tuberculosis, cuidadores, hermenêutica, pesquisa qualitativa, tuberculose.

## Abstract

**Objective.:**

To investigate the lived experience of family caregivers of persons with tuberculosis.

**Methods.:**

In this study, the method of hermeneutic phenomenology. Data were collected through online in-depth semi-structured interviews with nine family caregivers of TB patients. The obtained data were thematically analyzed to explain the concept of home care for TB patients through van Manen's 6-step methodology.

**Results.:**

After the thematic analysis, three main themes of caregivers' mental distresses, quality care stasis, and facilitated care were obtained from 944 primary codes and 11 categories.

**Conclusion.:**

Family caregivers of these patients suffer from mental distress. This issue affects the quality and ease of caregiving for these patients. Therefore, policymakers of this area should pay attention to the family caregivers of these patients and attempt to provide support; they should try to improve their quality of life.

## Resumen

**Objetivo.:**

Investigar la experiencia vivida por los cuidadores familiares de personas con tuberculosos.

**Métodos.:**

En este estudio se utilizó el método de la fenomenología hermenéutica. Los datos se recogieron mediante entrevistas semiestructuradas en línea con nueve cuidadores familiares de pacientes con tuberculosis. Los datos obtenidos se analizaron temáticamente para explicar el concepto de atención domiciliaria a los pacientes con TB mediante la metodología de 6 pasos de van Manen.

**Resultados.:**

Tras el análisis temático, se obtuvieron tres temas principales: angustia mental de los cuidadores, estancamiento de la atención de calidad y facilitación de la atención, a partir de 944 códigos primarios y 11 categorías.

**Conclusión.:**

Los cuidadores familiares de estos pacientes sufren de distrés sicológico. Este problema afecta la calidad y la facilidad con que se brinda el cuidado. Por lo tanto, los responsables de la política en salud deben prestar atención a los cuidadores familiares de estos pacientes para proporcionarles más apoyo, lo que redundará en una mejoría de su calidad de vida.

## Resumo

**Objetivo.:**

Investigar a experiência vivida por cuidadores familiares de pessoas com tuberculose.

**Métodos.:**

Neste estudo foi utilizado o método da fenomenologia hermenêutica. Os dados foram coletados por meio de entrevistas semiestruturadas online com nove cuidadores familiares de pacientes com tuberculose. Os dados obtidos foram analisados tematicamente para explicar o conceito de atenção domiciliar aos pacientes com TB utilizando a metodologia de 6 etapas de van Manen.

**Resultados.:**

Após a análise temática, três temas principais - sofrimento mental dos cuidadores, estagnação do cuidado de qualidade e facilitação do cuidado - foram derivados de 944 códigos primários e 11 categorias.

**Conclusão.:**

Os cuidadores familiares desses pacientes sofrem de sofrimento psíquico. Essa questão afeta a qualidade e a facilidade com que os cuidados são prestados a esses pacientes. Portanto, os responsáveis pela política de saúde devem estar atentos aos cuidadores familiares desses pacientes para dar-lhes mais suporte, o que resultará em uma melhora em sua qualidade de vida.

## Introduction

Despite 90 years of vaccination and 60 years of drug treatment, tuberculosis (TB) is still one of the leading causes of death by infectious agents.(1) In 2015, TB was one of the top ten causes of death worldwide.(1) Despite the efforts made to reduce the difficulties of the treatment process, TB is still a health problem, especially in developing countries.(2) According to a study, the average incidence of pulmonary TB was 7.5 per 100,000 people.(3) A study have shown a decline in the incidence of the disease in Iran between 2008 and 2017.(2) However, other study indicate an increase in the incidence of Extra pulmonary TB and highlight the need for special attention to this disease.(3) Since 1994, the World Health Organization has recommended the Directly observed treatment, short-course (DOTS) protocol for treating this disease.(4). Most infected people are treated with this outpatient method and have received home care from their families.(5) Therefore, family caregivers have an important role in adherence to treatment of patients. According to a study with a phenomenological approach in South Africa, social support contributed to adherence to treatment of these patients, and this support was better when the diagnosis and treatment of the disease were revealed to the patient's family members and friends.(6) A study in Iran also considered support from family effective in following the treatments for these patients.(7) The results of a relevant study in London also showed that social factors affected the delay in the diagnosis and treatment of TB.(8) On the other hand, a person with TB expects psychological support and guidance from his caregiver. In patient’s view, home is a proper place for rest and recovery.(5) 

Family caregivers of patients with TB have to do tasks such as monitoring the patient's adherence to the medication regimen; evaluating the side effects of medications; preparing a particular medication plan for the patient; checking his health condition, nutrition, and activity level; and ensuring the appropriateness of the schedule for referring the patients to health care providers.(9) These tasks and experiences are presented as challenges for caregivers that put pressure on them. On the other hand, living with a TB patient and providing care for him/her has created problems in families that are difficult to deal with.(6) Caregivers of patients with TB are ignored by the communities because they are close to these patients. This will affect the way the family takes care of these people.(10) These caregivers sometimes feel embarrassed, guilty, and angry.(11) It is noteworthy that whatever these family caregivers experience is private and are mentally affected by their conditions. Therefore, understanding them (caregivers) influences the quality of care they provide for these patients. The caregiver must be aware of the concept of what is important to patients and their families based on their own experiences, and they have a good understanding of them. Understanding the meaning of care is also essential for care providers.(12) On the other hand, most of the studies have examined the patients' lived experience.(6) (13)For example, a study examined the patients in South Africa with a phenomenological approach.(6) , or another case study investigated the lived experiences of a sick Spanish woman.(13) Therefore, considering the previous facts, valuable experiences of caregivers of these patients, lack of sufficient studies on these experiences in the world, especially in Iran, and dependability of family caregivers’ experiences on the context, qualitative research with a phenomenological approach helps to clarify the phenomenon of caring for the infected patients and gain a deep understanding of various aspects of this phenomenon. Since qualitative studies provide people with opportunities to express their views, values, and opinions(14) in the qualitative approach, reality emerges in the individuals’ minds. It exists in the context and can be examined through the inductive process.(15)

Researchers of present study are experienced in the field of TB patient care and interested in this subject. In fact, by conducting this qualitative study, they have reached the results based on the lived experiences of TB caregivers who are the main care providers for TB patients at home. Therefore, the present study was conducted to investigate the lived experience of family caregivers of TB patients using a phenomenological approach. It is hoped that nurses will use the knowledge gained in the cultural context in this regard to hold support programs for these patients. 

## Methods

Participants. This qualitative study was conducted using the hermeneutic phenomenological method that is descriptive-interpretive. The hermeneutic phenomenology approach and the van Manen method provide the possibility of achieving the lived experience of individuals from different perspectives. They also give a deeper understanding of the phenomenon under the study by interpreting the perception and lived experiences of numerous individuals and determining the shared features of the phenomenon.(16) Participants were selected through purposive sampling method from family caregivers of tuberculosis patients referred to Shiraz Pulmonary Diseases Center, which is the largest city and medical hub of southern Iran. The inclusion criteria in the study were: 1. Non-professionals who care for a sick family member with a diagnosis of TB. 2. Rich experiences in this regard and ability to communicate thoroughly. 3. Willingness to present their experiences to the researcher. 

Data Gathering. According to inclusion criteria 12 participants invited for in-depth interview. Two of them dropped out due to lack of time attending the interview and one for unfavorable health status. So, 9 participants attended for in-depth interview. After interviewing nine participants, the researchers concluded that deep and rich information had been obtained and that the data had been saturated. The researcher (RD, Ph.D candidate in Nursing) interviewed each of the nine participants individually, using a semi-structured in-depth interview by online video calls from June 2, 2020 to November 1, 2020, for 30 to 70 minutes. They asked open-ended questions (In the fact that you have been caring for your tuberculosis patient for some time, please explain your valuable experiences during this time; Would you explain one day of caring for tuberculosis patient from morning to evening?; When you hear about “caring for tuberculosis patient”, how do you feel?; When you hear about “caring for tuberculosis patient”, how you think about it?; When you hear about “caring for tuberculosis patient”, how you could describe it?; and: How “caring for tuberculosis patient” look like?) and probing questions (Would you explain more?; Would you narrate it more vividly?; and: Would you bring me an example?). Also, the rest of the questions were asked flexibly and according to the participants’ answers. The first two interviews considered as pilot testing of the questions and prompts, which completed for the next interviews. The first two interviews were discarded then. Interviews were conducted via online video calling and with the participant's permission recorded by a recorder. Then were transcribed word by word immediately after each interview; data collection continued until rich, in-depth, and relevant data emerged and the researchers concluded that adequate data had been collected and no new data would be obtained and data saturation had occurred. The final transcript returned to the participants for feedback and double checking. Then, the data were used for analysis. There was no drop out and no need for interview repetition. 

Ethical considerations. This research was approved by the Ethics Committee of X University of Medical Sciences (ethics confirmation number: X). All participants completed informed written consent to participate in the study. Also, the researcher guaranteed anonymity and confidentiality because the interviews were to be presented with a general perspective and did not focus on individual viewpoints. 

Data Analysis. Using the van Manen interpretive method, we analyzed the lived experiences of family caregivers of people with TB using open coding through MAXQDA2018 software. The researcher considered six methodological themes as a practical approach in conducting this interpretive phenomenology: 1. turning to the nature of the lived experience of TB caregivers while dealing with them was interesting for the researchers. Then, 2. Lived experience of the patient caregivers was investigated. At this stage, the researcher selected caregivers with rich experience, interviewed them, recorded, and transcribed their conversations word by word. 3. In the third phase, the researcher reflected on the essential themes which characterize the phenomenon. 4. Describing the phenomenon through the art of writing and rewriting was the fourth step. In this phase, the researcher wrote the necessary topics of the interview in the form of a story with a rich description of the caregivers' experience of caring for TB patients. 5. Maintaining a strong and oriented relationship with the phenomenon was the next step, in which the question "What is the lived experience of TB caregivers?" was considered. 6. In the last phase, balancing the research context by considering the parts and whole was considered, and finally, attention and orientation towards the nature of the lived experience of caregivers of TB patients. In this regard, the researcher maintained the relationship between different parts and the whole study in her mind. (17) (18) 

Rigor. The researcher used Lincoln and Guba criteria (19) to evaluate the robustness of the data. To increase the credibility, prolonged engagement for five months and eight days in the field was used. Peer debriefing was performed through repeated reviews of the research team and relevant professors. The researcher received feedback from the participants in the form of reviewing the results, codes, and their compliance with the views of the four participants. To enhance the transferability, we made an attempt to state the participants' sentences in the continuation of each theme clearly and completely. To increase dependability, we used the participants' triangulation by selecting individuals with different demographic characteristics. Attempts were made to make the results confirmable. Before giving their explanations, the participants did not read the opinions of other participants about the care of TB patients; they were unaware of their views, so the data were objective. The researcher addressed the concept of essence in phenomenology to achieve a perception of caring for patients with TB, appropriate to the research topic, by having an open view of the caregivers' lived experience. Their opinions were heard and recognized. (18)The researchers made reflection during the research process by being aware of their own thoughts and made regular efforts to consider their thoughts and actions to reduce bias in collecting, analyzing, and interpreting the data. 

## Results

The details of the participants’ characteristics are presented in Table 1. After thematic analysis, three main themes were obtained from 944 primary codes and 11 classes. They included caregivers' mental distresses, quality care stasis, and facilitated care (Table 2). Lived experience of family caregivers of patients with TB means mental distress and different quality of care (stasis and facilitated). 

**Table 1:** Demographic Information of the Participants

Participant	Gender	Relationship with the patient	Age (in years)	Marital status	Occupation
1	Female	Daughter	23	Single	Housekeeper
2	Male	Spouse	27	Married	Self-employment
3	Male	Son	54	Married	Self-employment
4	Female	Daughter	35	Married	Self-employment
5	Female	Mother	68	Widowed	Housekeeper
6	Male	Brother	22	Single	Self-employment
7	Female	Sister	42	Married	Housekeeper
8	Female	Mother	33	Married	Housekeeper
9	Female	Spouse	56	Married	Housekeeper

**Table 2:** Themes, Categories, Subcategories

Themes	Categories	Subcategories
I. Caregivers' mental distresses	1. Perception of TB as a confusing disease	Caregivers' confusion about tuberculosis
		Hesitation about health care providers’ competence
		The fear of tuberculosis
		Fear of incurability before diagnosis
	2. Mental and psychological distress of caregivers during illness	Caregivers' mental rumination
		Expressing frustration by the caregiver
		Caregiver’s anger
		Caregivers’ fear and worry
		Caregivers’ discomfort
		Feeling embarrassed due to having tuberculosis
II. Quality care stasis	1. Carrying the burden of care alone	Being single caregiver
		Lack of emotional support from relatives
	2. Unease of unwanted cases	Coincidence of TB with the prevalence of COVID -19 disease
		Involvement of family feelings due to the coincidence of TB with other diseases
		Co-occurrence of TB with other disease
	3. The paradox of observing and not observing standard precautions	Failure to follow the standard precautions of tuberculosis
		2. Observing precautions related to tuberculosis
	4. Non-acceptance in society	The society's negative views about tuberculosis
		Concealing diagnosis
III. Facilitated care	1. Facilitating role of the COVID crisis for caregivers	Improvement of treatment conditions
		Facilitation of mandatory observance of TB precautions
		Limited commutation
	2. Occurrence of adaptive behaviors of caregivers with tuberculosis	Caregivers' preliminary knowledge about TB
		Disclosure of diagnosis
		Resilience of caregivers
		Attempts to acquire basic knowledge about tuberculosis
	3. Faith in God and hope for the future	Having faith in God during the period of illness
		Satisfaction with uncontamination of caregiver
		Caregiver’s increased hope of not being alone in diagnosing tuberculosis
		Hope for a good prognosis
	4. Being supporter of patients with tuberculosis	Perceived supportive behaviors by the treatment system
		Spiritual support from friends
		Strong family support in patient care
		Caregivers’ efforts to improve their family and themselves’ mental condition

Theme I: Caregivers’Mental DistressesThe theme of caregivers’ mental distress indicates that there have been factors creating mental confusion among caregivers' mental distress indicates that there have been factors creating mental confusion among caregivers while caring for their TB patients. The categories extracted for this theme were the perception of TB as a confusing disease and the mental and psychological distress of caregivers during illness. 

*Perception of TB as a confusing disease.* This category reflects the ambiguous nature of TB from the caregivers' point of view, which has subcategories such as caregivers' confusion about tuberculosis. In this regard, one of the participants said: *The exact onset of my mother's illness was not known. In fact, we did not know anything about TB at the time and did not know what to do (Participant 3).* The hesitation about health care providers’ competence is another subcategory of perception of TB as a confusing disease category. One participant says: *You know, when I was in the hospital, everyone was asking me how I was taking these drugs. Their knowledge was more than mine, but they did not know their knowledge about this disease was not enough. (Participant 8).* The fear of tuberculosis was another subcategory of perception of TB as a confusing disease category. This fear included fear of disease and the fear of family members getting tuberculosis. About this, participants said: *secret pains that only you know, not anybody else. Horrible pains in all your body. (participant 9).* The fear of incurability before diagnosis was another concern of caregivers. These fears became apparent by the caregivers’ numerous visits to physicians to discover and diagnose their fear of incurable diseases: *We were afraid of probable hazards; my sisters and my mother were scared. Not knowing what is good for this disease, they feared this disease would lead to her death. (Participant 6)*

*Mental and Psychological Distress of Caregivers during Illness.* The second main category of the theme of caregivers’ mental distresses was related to the mental and psychological distress of caregivers during the illness, which includes the subcategory of the caregivers’ mental rumination, expression of frustration by the caregiver, Caregiver’s anger, Caregiver’s fear and worry, Caregiver’s discomfort, and a feeling embarrassed due to having tuberculosis. Existence of dual feelings, hesitation, remorse for not telling others about the diagnosis, repeated uncertainties, and inability to express the feelings expressed under the subcategory of caregivers’ mental rumination: *Maybe, it would have been much better if I had told others about the diagnosis, but I did not say, and it cannot be said now. (Participant 4).* One caregiver talked about expressing frustration: *I'm no longer living my life; I'm no longer living. It’s all nonsense. (participant 5).* Fear and worry were the other emotions expressed by caregivers: *They said he would get well. Well, I had hope, but I used to say what if my father did not get well, or what if he got worse. Or I had heard that this medicine would harm the liver; then, I thought if he did not take the medicine to cure one organ, would it cause another organ in his body to get infected? (Participant 1).* Another participant states: *They said he would get well. Well, I had hope, but I used to say what if my father did not get well, or what if he got worse. Or I had heard that this medicine would harm the liver; then, I thought if he did not take the medicine to cure one organ, would it cause another organ in his body to get infected? (Participant 1).* One caregiver expressed embarrassment about tuberculosis: *I don't know if this disease is embarrassing or something like that or ugly (Participant 4).*

Them II: Quality Care Stasis. Caregivers mentioned many things in their experiences that could decrease the quality of care and make it difficult. The sub-categories of this theme were the Carrying the burden of care alone, unease of unwanted cases, the paradox of observing and not observing standard precautions, and non-acceptance in society. 

*Carrying the burden of care alone* Six of participants complained about being a single caregiver and lack of emotional support from relatives in caring for their patients, which is an obstacle to quality care. One of them about being single caregiver said: *I was doing the hospital work alone because my sister, mother, and father were burnt in an incident, and my brothers were young, too. I was under high financial pressure, and it was all on me. (Participant 6).* Another participant said about Lack of emotional support from relatives*: In this disease, I was involved more than the other children. I did all the works by myself; I took him to the hospital myself, but the others helped less during this time; however, I was the main person who did all the care (Participant 4).*

*Unease of unwanted cases.* The experience of unforeseen events during TB has sometimes made it difficult for caregivers to tolerate the disease. These cases have also put the family under pressure to provide better care. Coincidence of TB with the prevalence of COVID-19 disease, Involvement of family feelings due to the coincidence of TB with other diseases, and co-occurrence of TB with other disease are among the lower classes in this main category: *I'm terrified since the emergence of Coronavirus; that is, if one of the children coughs, I feel I’m dying. I feel dead since I fear it may be COVID-19 (Participant 9); Since the beginning of the pandemic, my father was in hospital. We were afraid they might get infected with the Corona virus since they were there for their TB. We weren’t scared for our brothers because they were young. Our main fear was for our parents. We observed all the protocols; however, when a patient with COVID-19 walked past us, we were scared. It was dangerous (Participant 1).* One participant talked about the co-occurrence of tuberculosis with other diseases: *Later, when the family was like this, their burn in the accident made the situation more difficult. I was stuck in my sister's problem that the fire happened and made the situation worse (Participant 6).*

The paradox of observing and not observing standard precautions. The observation of the standard precautions related to TB was presented in a contradictory way by the caregivers. A participant who also had TB talked about the experience of observing precautions related to tuberculosis in this way: No one approached him. He was one meter or two away from us. We found out that he had the disease. We separated his room and did not have much contact with him. We separated him, washed, cleaned, and disinfected. When he was sick, the child wouldn’t come to our house. My sister always wore a mask (Participant 6). In contrast, some caregivers' experiences showed failure to follow the standard precautions of tuberculosis for some reasons: I did not know before that he had this problem. I was very close to him. I think they told me to keep the distance, but the truth is that I have been involved in it for 2-3 years now. I did not observe at that time. It’s very funny if I observe now. (laughter) Why should I lie? I did not separate his dishes either. I said that he would be more disturbed if I attempted to separate his dishes (Participant 8). 

Non-acceptance in society. The last subcategory was the main category of quality care stasis in a non-acceptance of society. From the caregivers' point of view, the society's negative views about TB and concealing diagnosis were two subcategories mentioned by the caregivers. An example of the participants' statements about the negative view of society by caregivers is as follows: The one whose mother was infected is not told about the diagnosis, but they say the whole family should take the test every couple of years. It was like this that instructions that were informing and efficient are not available. I hope there comes a day when doctors say TB and HIV are similar to cold, or say: you are clean if you take this test. I wish that and pray to God for that. I hope we see a day when this disease is treated as cold in our country (Participant 9). Here is what a participant said about the fear of judgment following the disclosure of a diagnosis:For example, in our neighborhood, all the people are like this. When they hear a word, they say the opposite and spread it. And here, everyone knows about each other. Therefore, we decided not to tell anyone because we didn’t want them to backbite us or say bad words behind us (Participant 1). 

Facilitated care The third theme obtained from the analysis of the lived experience of participants is facilitated care. Categories related to the theme including Facilitating role of the COVID crisis for caregivers, Occurrence of adaptive behaviors of caregivers with tuberculosis, faith in God and hope for the future, being supporter of patients with tuberculosis, and ease of care after a challenging onset. 

Facilitating role of the COVID crisis for caregivers. The three subcategories obtained were improvement of treatment conditions, facilitation of mandatory observance of TB precautions, and limited communication. Also, caregivers experienced numerous satisfactory experiences regarding the role of Corona virus crisis facilitation: The prevalence of Corona pandemic was not that much annoying for us. He had to take his medication on time, so I had to take his medications to school. They were three or four. Some of them had to be taken with short intervals. Yes, Corona was in our favor in places because I could give his medicine regularly (laughs) (Participant 8). 

Occurrence of adaptive behaviors of caregivers with TB. Over time, caregivers experienced adaptive behaviors that helped them achieve quality care for their patients. Subcategories of this category included the caregivers' preliminary knowledge about TB, disclosure of diagnosis, resilience of caregivers, and attempts to acquire basic knowledge about tuberculosis. Caregivers' preliminary knowledge about TB had made caregivers more adaptive to the disease. One the caregivers stated: I mean, I found out that TB was a dangerous infectious disease. I noticed that my mother went to the lady who died of the same disease almost a year ago. Because, At the same time, the same lady was visiting my mother, my mother got emotional and got the disease. (Participant 3). Disclosure of diagnosis of tuberculosis to others was another factor in the occurrence of these adaptive behaviors. In this regard, a caregiver said: Our landlord who is with of us could see what a bad cough he was having. I had told my mother and sister what the problem was because they kept asking. I had also told my sister-in-law that she had tuberculosis, but her aunt, who lives in Shiraz, was coughing. When she asked what had happened, I told her it was TB (Participant 6). Resilience of caregivers was another important subcategory that most participants cited in their experiences: The children had accepted him, too. We did not have a big problem, so we did not avoid him. They had gotten along with their father. When I went for the test, I came and said: “Mom, do not be afraid. I got TB and, there is nothing to worry about; but, what could I do? Children said: you are not afraid. I said black will take no other hue. Why should I be afraid? (Participant 9). Attempts to acquire basic TB knowledge were reported as another factor in increasing caregivers’ adaptation: Sometimes, Mr. A, whom I would text him or go to him to get medicine, would say: No, he will be okay. Well, they have more knowledge about the disease than us (Participant 8). 

Being supporter of patients with TB. The other main category of facilitated care is related to the patient support experience of a patient with TB that includes subcategories of perceived supportive behaviors by the treatment system, spiritual support from friends, strong family support in patient care and caregivers’ efforts to improve their family and themselves’ mental condition. The caregiver's attempt to improve his or her mental state or family is similar to using the psychological advice of a patient-supporting subcategory, which is contagious. A caregiver said: I took my parents to a psychologist to convince them that this was not dangerous for them. I have a psychologist friend, but I talked to him not as a psychologist, but as a friend for 10 minutes every night. Well, I could do many things. I was getting relieved, and I knew what I was supposed to do tomorrow (Participant 7). One participant talked about cohesive patient support: I took his blood test for liver and kidneys every two or three weeks because they said the disease has side effects on other organs. I wanted to make sure he did not have any other problems and could do his routines. I was asking because I was so obsessed. I was asking a different doctor. I was asking as many people as I could (Participant 4). 

Being at ease of care after a challenging onset. The subcategories related to this main category are the early difficulties of the disease, and Improvement of the conditions by the start of treatments. Subcategories related to the early difficulties of the disease included the patient's constant objections to caregivers during care, the physical problems of the primary caregiver, mental health problems, financial problems, and disruption of life routines. During the disease in the family, some participants expressed the patient's numerous objections to the early difficulties of the disease: One of them told me to take him to the hospital to drain his neck infection. He said, 'No, I can't stand it. We insisted a lot; we said let's go. He said: I have such bad experience in the hospital that I am not willing to go there again (Participant 9). Some caregivers also reported financial problems in the family: I had to spend most of the money I had saved. I borrowed it from here and there. It was a high pressure on me (Participant 6). Improving the condition with the start of treatment was another subcategory, which included subcategories such as improvement of the physical condition of caregivers, caregivers' satisfaction, improvement of the patient's situation, and meeting the family's financial needs at the time of illness. One caregiver talked about reducing the time of night awakenings as conditions improved: The nights before he took the pills, he would wake up three times and go to the bathroom. The first night was the same, but from the second night onwards, when he took the pills, it got much less (Participant 3). The caregivers' sense of satisfaction with the continuation of the care was apparent in their conversations: I mean, there is no problem at all, just a slight pain. My dad no longer sleeps in bed at all, and they are all out; everything is normal (Participant 1). About meeting the family's financial needs, one caregiver said: Thanks God, they are giving us free medicine now (Participant 2). 

## Discussion

The main purpose of this study was to examine, describe, and interpret the lived experience of family caregivers of TB patients to gain an insight into the phenomenon that the caregivers mentioned in their words. The themes were the answer to the question: what is the lived experience of caring for TB patients? To answer this question, we can say that caring for TB patients at home includes the three themes of caregivers' mental distresses, quality care stasis, and facilitate caring. According to the results of the present study, factors such as confusion, fear of illness, frustration, anger, and shame lead to ambiguity and psychological distress of the caregivers. These factors ultimately cause mental disorders in family caregivers of TB patients. A study of 35 family caregivers of children with TB in Mozambique also revealed that the diagnosis was associated with adverse reactions such as pity, fear, worry, anger, and sadness.(20) Phenomenological studies on caring the relatives of TB patients in India have also reported mental and psychological fatigue such as hatred, anger, and impatience.(21) A study of content analysis on TB patients and their family caregivers in Indonesia showed that TB stigma associated with psychosocial problems, including feelings of shame and social exclusion, was influenced by fear of transmitting the disease and the others’ blame. (22) A qualitative study of nurses caring for TB patients in South Africa conducted the themes of fear, anxiety, stress, and risk of getting infected. (23) Therefore, several studies have shown the confusion, mental and psychological pressures in caregivers of TB patients. It is also necessary to pay attention to them and provide psychological support through various means such as referral to psychologist or establishing mental counseling centers for TB patients and their caregivers. 

Another theme was the quality of care stasis, which included a sense of loneliness in care, unease of unwanted cases, the paradox of observing and not observing standard precautions, and non-acceptance of society. In the study on caregivers of Indian patients, meeting the environmental health needs of patients has been obtained from one of the subcategories. (21) This attempt is in line with the category of observance and non-observance of standard precautions in the present study. In a content analysis study on patients with TB and their family caregivers in Indonesia, patient isolation was one of the subcategories. (22) One of the duties of the family is to observe the health protocols and keep the environment clean. Also, the caregiver must make sure the environment is clean and maintain standard precautions. (21) A study of children with TB in Mozambique showed a lack of adherence to treatment and care in one-fourth of patients. (24) According to Mindu’s study, the patterns of delayed care search related to the official health care system and follow-up treatment; therefore, significant efforts should be made to inform people about the long and complex nature of TB diagnosis and treatment, so that the community adapts with the formal system despite its tendency for care alternatives. (20) 

One study found that Indonesian families and patients considered TB a frightening, severe, and dangerous disease(22) It also revealed that this affected the adherence to treatment and quality of patient care. The support from family members is vital for the patient's care and following treatments, so the main reason for discontinuation of treatments in some patients is the lack of support from family members. (21) Hence, family support is crucial in the continuation or otherwise of quality care in TB patients. 

The last theme obtained in the present study was the facilitated care, one of the subcategories of which was the facilitating role of the Corona crisis for caregivers because the observance of standard precautions and social isolation for TB patients and their families leads to their social isolation. Thus, as soon as others know about their diagnosis, they avoid these patients because of their disease, and exclude them from their social life. (22) However, social distancing and standard precautions created by the Corona pandemic make it easier for families of TB patients to follow through, and there is no need to explain the reason for standard precautions to other people who are in touch with them. In this way, social exclusion due to the fear of transmitting TB and the others’ blame was reduced because, in the current situation, all members of society should observe social distancing and standard precautions. 

The occurrence of adaptive behaviors of caregivers with TB was another facilitator of care. A phenomenological study on Ethiopian patients with TB also showed a positive effect of the patients' adaptation to family and the others' reactions on the process of their care and recovery. (25) In a study by Rakhmawati, (22) Indonesian caregivers and TB patients also used different strategies to adapt to the TB stigma. These adaptive behaviors reflect the value of families in maintaining the relationships and the intimacy and loyalty of other family members to protect and help each other. (22) Providing psychological support to facilitate the caregivers' adaptation to this situation and maintain their productivity and active care is crucial. (23) Faith in God and hope for the future were other facilitators of care found in the caregivers' words. Disappointment is peculiarly predictable in patients with refractory TB that requires special attention. (25) 

Another facilitation of care is supporting TB patients. Psychosocial support from family, friends, and the community is crucial for the treatment of these patients. It helps to overcome the fear of the disease and its complications, so home care facilitates it. (6) (25) Infected patients are sometimes kept in nursing homes, which prevents them from receiving emotional, social, and physical support from their families; therefore, it harms the patients' recovery. A person with TB should not be deprived of social and psychological support from society. In this respect, awareness about the disease and its consequences at the community level is necessary. Family caregivers play a significant role in protecting the patient against treatment complications and in monitoring treatment and maintaining a healthy environment. However, caregivers themselves need financial, psychological, and social support, and the presence of a mental health counselor is necessary to provide emotional and psychological support to patients and their family caregivers. (21) The results of a study on TB patients and their caregivers in Indonesia indicated their need for support from health care providers to overcome the stigma of the disease. (22) In the present study, the subcategory of ease of care after a challenging onset refers to the financial, mental, and physical problems of caregivers. This subcategory attracts the health officials and policymakers' attention to providing solutions to support the patients' family caregivers. Studies also show that counseling, psychosocial support, and awareness about the disease are efficient ways to help TB patients and their families to cope with the disease, so efforts should be made in this regard. (21) Socio-cultural support, formation of support groups, provision of information, and strategies to improve the quality of life for these patients and their families are recommended. (6) 

This study has many Implications such as providing insights into community members’ perceptions of TB and how this affects health-seeking behavior and quality of life. The participants mentioned stigma and discrimination within the community primarily due to the perceived TB. The expressed experiences highlight the interplay of factors at individual, household, and community levels and how they affect TB-related health-seeking behavior, diagnosis, and treatment. 

## Conclusion

Finally, it can be said that the caregivers' mental distress, quality care stasis, and facilitated care have been among the experiences of family caregivers of TB patients. The authors hope that the results of the study will contribute to the planning of managers and policymakers of this disease to provide solutions for these families to overcome their daily challenges and grant quality of life for these caregivers, so that due to their quality of life improvement, TB patients’ life quality improves. 

One of the limitations of the present study was the impossibility of face-to-face interviews due to the conditions caused by the Corona pandemic, in which online video calls were used to address this issue. Also, in this study the collection of data was just through individual interviews and making use of other data collection methods could enrich the results of this study. Another limitation of this study was that the participants were only from one center and the selection of them from other centers in the country could improve the generalizability of the findings, so more qualitative and quantitative research on larger samples in other places and cultures is needed to investigate the lived experience of family caregivers of tuberculosis patients. 
